# RNA Sequencing-Based Transcriptome Analysis of Liver in Laying Hens Supplemented with Dietary Probiotic *Bacillus* Species and Prebiotic Yeast (*Saccharomyces cerevisiae*) Cell Walls

**DOI:** 10.3390/vetsci12090822

**Published:** 2025-08-27

**Authors:** Ala E. Abudabos, Zafar M. Hakami, Ali R. Al Sulaiman, Riyadh S. Aljumaah, Valentino Palombo, Mashael R. Aljumaah, Mariasilvia D’Andrea, Abdulrahman S. Alharthi, Rashed A. Alhotan

**Affiliations:** 1 Department of Food and Animal Sciences, College of Agriculture, Tennessee State University, Nashville, TN 37209, USA; 2 Department of Animal Production, College of Food and Agriculture Sciences, King Saud University, P.O. Box 2460, Riyadh 11451, Saudi Arabia; 437105818@student.ksu.edu.sa (Z.M.H.); rjumaah@ksu.edu.sa (R.S.A.); abalharthi@ksu.edu.sa (A.S.A.); ralhotan@ksu.edu.sa (R.A.A.); 3 Environmental Protection Technologies Institute, Sustainability and Environment Sector, King Abdulaziz City for Science and Technology, P.O. Box 6086, Riyadh 11442, Saudi Arabia; 4 Department of Agricultural, Environmental and Food Sciences, Università degli Studi del Molise, Via de Sanctis SNC, 86100 Campobasso, Italy; valentino.palombo@unimol.it (V.P.); dandrea@unimol.it (M.D.); 5 Department of Botany and Microbiology, College of Science, King Saud University, P.O. Box 2455, Riyadh 11451, Saudi Arabia; maljumaah1@ksu.edu.sa

**Keywords:** dietary supplements, laying hens, liver transcriptome sequencing, prebiotic yeast cell wall, probiotic *Bacillus* species

## Abstract

To investigate the impacts of dietary *Bacillus*-based probiotics and yeast-derived prebiotics on the hepatic transcriptome profile, 500 Hisex White laying hens were randomly allotted into five dietary treatments from 37 to 52 weeks of age: control; control + *Bacillus subtilis*; control + *Bacillus subtilis* and *Bacillus licheniformis*; control + *Bacillus coagulans*; and control + *Saccharomyces cerevisiae* yeast cell wall. Transcriptome analysis revealed a substantial number of differentially expressed genes exclusively between the control and prebiotic groups, identifying 2221 genes (FDR ≤ 0.05), with 980 genes upregulated (log_2_ fold change 0.69 to 24.62) and 1241 downregulated (log_2_ fold change −0.74 to −26.46). The top 10 upregulated KEGG pathways included protein export, glycerophospholipid metabolism, tryptophan metabolism, amino acid biosynthesis, alanine, aspartate, and glutamate metabolism, cofactor biosynthesis, propanoate metabolism, ABC transporters, 2-oxocarboxylic acid metabolism, and protein processing within the endoplasmic reticulum. In contrast, the most prominently downregulated pathways encompassed fructose and mannose metabolism, hedgehog signaling, PPAR signaling, Notch signaling, GnRH signaling, cell adhesion molecules, cytokine–cytokine receptor interactions, apelin signaling, glycosaminoglycan degradation, and RIG-I-like receptor signaling. These findings advance understanding of the hepatic transcriptomic response to yeast-derived prebiotics and identify key molecular pathways that could be targeted to enhance metabolic function in laying hens.

## Simple Summary

The poultry industry has long relied on antibiotic growth promoters (AGPs) to enhance growth, feed efficiency, and disease resistance. However, rising concerns over antimicrobial resistance and residues in poultry products have driven interest in safer alternatives. Nutritional strategies, particularly probiotics and prebiotics, are being explored as potential replacements. This study investigated the effects of *Bacillus*-based probiotics and yeast-derived prebiotics on the hepatic transcriptome of laying hens using RNA sequencing. A total of 500 Hisex White hens were assigned to five dietary treatments from 37 to 52 weeks of age. Significant transcriptomic changes were observed only in the prebiotic group compared with the control, identifying 2221 differentially expressed genes: 980 upregulated and 1241 downregulated. Upregulated pathways included protein export, glycerophospholipid metabolism, amino acid biosynthesis, cofactor biosynthesis, propanoate and 2-oxocarboxylic acid metabolisms, and protein processing within the endoplasmic reticulum. Conversely, downregulated pathways involved carbohydrate metabolism, key signaling pathways (hedgehog, peroxisome proliferator-activated receptors, Notch, gonadotropin-releasing hormone), immune interactions, and glycosaminoglycan degradation. These findings provide new insights into how yeast-derived prebiotics modulate liver gene expression, highlighting molecular pathways that may enhance metabolic function and support sustainable poultry production.

## 1. Introduction

The poultry sector has traditionally relied on antibiotic growth promoters (AGPs) to boost growth performance, enhance feed utilization, and minimize disease occurrence [1]. Nevertheless, increasing apprehension regarding antimicrobial resistance and antibiotic residues in poultry products has led to a shift toward safer and more sustainable approaches [2]. As a result, nutritional interventions such as probiotics and prebiotics are being actively investigated as viable replacements for AGPs. In particular, probiotic strains like *Bacillus subtilis*, *Bacillus licheniformis*, and *Bacillus coagulans* have shown considerable potential, while the yeast cell wall of *Saccharomyces cerevisiae* is being studied for its notable prebiotic effects [3].

*Bacillus subtilis* and *Bacillus licheniformis* are widely researched probiotics valued for their resilience and capacity to form endospores, enabling them to withstand extreme conditions, including the low pH of the gastrointestinal tract [4]. These species synthesize a range of digestive enzymes, such as proteases, amylases, and cellulases, that support more efficient breakdown and assimilation of nutrients in the host organism [5]. Their enzymatic actions also lower intestinal content viscosity, fostering a more balanced and resilient gut environment [6]. Moreover, they contribute to gut health by generating antimicrobial compounds like bacteriocins and lipopeptides, which suppress harmful bacterial populations, thereby supporting a balanced gut microbiome and lowering the risk of gastrointestinal infections [7]. *Bacillus coagulans*, though not as extensively researched as other *Bacillus* species, shows significant potential as a probiotic in poultry nutrition [8]. Its notable capacity to produce lactic acid and various organic acids contributes to the acidification of the intestinal environment, thereby suppressing the proliferation of harmful microorganisms [9]. Furthermore, this bacterium supports gut function by stimulating the formation of short-chain fatty acids (SCFAs), which contribute to colonocyte energy metabolism, support microbial homeostasis, and modulate immune responses [10].

The cell wall of *Saccharomyces cerevisiae* exhibits a highly intricate composition, predominantly consisting of mannoproteins, β-glucans, and chitin. Each of these constituents contributes distinctly to the structural integrity and biological activity of the yeast cell wall, which has been recognized for its potential health-promoting effects in animal nutrition [11]. Mannoproteins, located predominantly in the outermost layer of the yeast cell wall, function as glycoproteins capable of binding pathogenic bacteria, thereby inhibiting their adhesion to intestinal epithelial cells and decreasing the likelihood of infection [12]. β-glucans, a class of polysaccharides involving glucose polymers, have been extensively studied for their role in immune modulation, notably through activation of immune cells such as macrophages, neutrophils, and natural killer cells [13]. Although present in relatively smaller amounts, chitin and its derivatives like chitosan also contribute beneficially by exhibiting antimicrobial activity and influencing the gut microbial balance [14]. Collectively, these bioactive components endow the yeast cell wall with strong prebiotic properties that can improve both health and productivity in poultry [15].

In addition to their established effects on gut health, these dietary supplements have also demonstrated potential hepatoprotective properties. The liver, being a central organ in metabolism and immune regulation, is closely influenced by gut-derived signals, such as microbial-associated molecular patterns (e.g., lipopolysaccharide, peptidoglycan), microbial metabolites (e.g., short-chain fatty acids, secondary bile acids), and host-derived mediators (e.g., cytokines, chemokines), through the gut–liver axis [16]. Dietary *Bacillus subtilis* supplementation has been shown to reduce hepatic oxidative stress and inflammation in broilers, potentially through modulating gut microbiota and lowering endotoxin translocation to the liver [17]; however, whether these effects extend to laying hens remains to be fully elucidated. Similarly, *Bacillus licheniformis* has been associated with improved hepatic antioxidant status in chicken, likely by stimulating the Nrf2/Keap1 signaling pathway and suppressing hepatocyte apoptosis and autophagy via modulation of related genes [18]. The *Saccharomyces cerevisiae* cell wall extract has also been reported to exert hepatoprotective effects through its immunomodulatory and anti-inflammatory properties, potentially reducing hepatic cytokine production and oxidative damage [19]. These findings support the hypothesis that these supplements not only improve intestinal health but may also confer hepatoprotective effects, underscoring the importance of investigating their influence on liver gene expression and metabolic pathways in poultry. Nevertheless, to the best of our knowledge, no hepatic transcriptomic studies have been conducted to examine the molecular and functional changes induced by supplementing laying hen diets with *Bacillus*-based probiotics and yeast-derived prebiotics as natural substitutes for AGPs. Consequently, this study aimed to evaluate and compare the impacts of these dietary supplements on the liver transcriptome of laying hens utilizing RNA-sequencing analysis, including the identification of differentially expressed genes and enriched biological pathways, thereby advancing the understanding of gut-liver interactions in poultry.

## 2. Materials and Methods

### 2.1. Experimental Birds and Dietary Treatments

The animal study protocol was approved by the Ethics Committee of King Saud University, Riyadh, Saudi Arabia (KSU-SE-21-38).

A total of 500 Hisex White laying hens, aged 37 weeks and exhibiting alike body weights averaging 1.64 kg and a laying performance of approximately 94.2%, were housed in 25 floor-based cages, each with dimensions of 1.9 by 2.0 m. Bird placement across cages was randomized to control for potential confounding effects related to cage location and housing conditions. The hens were then assigned to five experimental groups following a completely randomized design, with allocation performed utilizing a computer-generated randomization sequence. Each group consisted of five replications of 20 chickens. The experimental diets included the following: a basal diet with no additives (control); the basal diet plus *Bacillus subtilis* (DSM17299) at 1.1 × 10^8^ CFU/kg (probiotic-1); the basal diet plus a blend of *Bacillus subtilis* (DSM5750) and *Bacillus licheniformis* (DSM5749) in equal proportions, totaling 1.3 × 10^9^ CFU/kg (probiotic-2); the basal diet supplemented with *Bacillus coagulans* (DSM32016) at 1.0 × 10^9^ CFU/kg (probiotic-3); the basal diet with the addition of *Saccharomyces cerevisiae* yeast cell wall at 0.25 g/kg (prebiotic). All supplementation levels were based on manufacturer recommendations and were thoroughly mixed into the feed, with final concentrations verified analytically. The feeding trial spanned 16 weeks, concluding when the hens attained 52 weeks of age.

The clinically healthy hens were purchased from a commercial chicken farm with a verified history of high biosecurity standards and vaccination compliance. Before initiating the experiment, the chickens underwent an adaptation period lasting two weeks, during which their diet was gradually shifted from the standard feed to the experimental formulations. The control diet, primarily composed of corn and soybean meal, was prepared based on the nutritional guidelines established by the NRC [20] to fulfill the nutritive necessities of laying hens. The diet formula composition is displayed in Table S1. The nutritional value of the feed constituents utilized for the formulation was analyzed by applying standard AOAC procedures [21]. The birds were accommodated in an environmentally controlled setting, maintaining an average temperature of 22 ± 2 °C and relative humidity of 55 ± 5%, under a 16 h light (15 lux) and 8 h dark photoperiod. During the experimentation, the hens were managed in compliance with established husbandry and biosecurity protocols and granted unrestrained access to the experimental mashed diets and fresh water. The hens were observed at least twice daily for general health status, behavior, feed and water intake, and signs of distress or illness. No unexpected or adverse events were observed during the course of the study. The hens maintained general good health, with no signs of morbidity or mortality attributable to the experimental diets or environmental conditions.

### 2.2. Sample Collection and RNA-Seq-Based Transcriptome Analysis

At 52 weeks of age, ten birds of average body weight were randomly chosen from each experimental group, humanely euthanized, and subjected to dissection. The entire liver was excised, and roughly 1 g of liver tissue was instantly immersed in RNAlater RNA stabilization reagent (Thermo Fisher Scientific, Waltham, MA, USA). Specimens were then kept at −80 °C pending further processing. Subsequently, total RNA was isolated from 100 mg of frozen liver tissue utilizing the PureLink™ RNA Mini Kit (Invitrogen, Carlsbad, CA, USA) as per the manufacturer’s protocol. RNA quantity and purity were assessed spectrophotometrically using a NanoDrop 2000 (Thermo Fisher Scientific, Waltham, MA, USA), while RNA integrity was evaluated via 2% agarose gel electrophoresis and with the RNA 6000 nano kit on the Bioanalyzer 2100 system (Agilent Technologies, Santa Clara, CA, USA). The purified, high-quality RNA samples, with RNA integrity number (RIN) values ≥7, were then stored at −80 °C for subsequent high-throughput sequencing.

RNA sequencing was conducted employing a HiSeq 2500 platform (Illumina Inc., San Diego, CA, USA). Briefly, strand-specific RNA-seq depleted ribosomal RNA was prepared utilizing the TruSeq stranded total RNA sample preparation kit with Ribo-Zero gold (RS-122-2301/RS-122-2302, Illumina). Additionally, transcriptome capture libraries were constructed utilizing the TruSeq RNA access library preparation kit (RS-301-2001, Illumina). Each library was adjusted to a concentration of 20 pM and sequenced on the instrument employing Illumina TruSeq V3 chemistry in 2 × 250 bp paired-end reads, involving cluster generation and a 50-cycle paired-end run. Image processing and base calling and quality scoring of the run were executed utilizing Illumina’s real-time analysis software (version 1.18.54), and the resulting data were demultiplexed and transformed into FASTQ input format utilizing Illumina’s bcl2fastq software (version 2.20.0). All procedures were carried out in accordance with the manufacturer’s instructions.

The raw sequence reads underwent quality assessment applying FastQC software (version 0.11.8). Adapter sequences and low-quality bases were then filtered out utilizing TrimGalore software (version 0.6.5), applying a Phred quality score threshold of 20 and retaining reads with a minimum length of 30 bp. Afterwards, high-quality reads were aligned to the *Gallus gallus* reference genome (GRCg7b) with Hisat2 software (version 2.0.4), followed by sorting of the aligned reads with SAMtools software (version 1.19). Differential gene expression between experimental groups was analyzed employing DESeq2 (version 1.42.0) software [22]. Genes exhibiting a Benjamini–Hochberg false discovery rate (FDR)-corrected *p*-value ≤ 0.05 and an absolute log_2_ fold change ≥ 1 were identified as significantly differentially expressed genes.

To elucidate the biological roles associated with the differentially expressed genes, functional enrichment analyses were conducted utilizing both Gene Ontology and Kyoto Encyclopedia of Genes and Genomes (KEGG) pathways through the database for annotation, visualization, and integrated discovery (DAVID, version 6.8). To ensure robustness of the findings, the analyses were further validated with DAVID v2021, which produced consistent enrichment patterns. Gene Ontology-based functional annotation of DEGs was categorized into two primary domains: Molecular Function and Cellular Component. For KEGG pathway analysis, the Dynamic Impact Approach was employed. This method evaluates biological pathways by computing two key parameters: overall impact, reflecting the relative importance or significance of a pathway, and flux, indicating the direction of regulatory changes (e.g., upregulation, downregulation, or no significant change), based on gene fold-change values, as previously described [23]. In brief, the analysis incorporated the entire dataset encompassing Entrez gene IDs, FDR ≤ 0.05, fold-change, and *p*-value ≤ 0.05. Pathway impact and direction were subsequently computed only for KEGG terms with at least 30% representation in the microarray relative to the annotated genome, interrogating the full KEGG database. Enrichment of Gene Ontology terms and KEGG pathways was deemed statistically significant if the gene count exceeded two and the Benjamini–Hochberg corrected *p*-value was below 0.05.

Using the list of differentially expressed genes, a protein–protein interaction network analysis was constructed with the STRING database [24]. The analysis of protein–protein interaction facilitates the identification of fundamental organizational principles within functional networks. To identify potential hub proteins within the network, both local (Degree method) and global (Radiality method) centrality analyses were conducted employing the Cytoscape software (version 3.9.1) plugin cytoHubba [25]. Specifically, the local approach ranks hubs based on direct node-neighbor connections, while the global method considers interactions between each node and the entire network. The top ten hubs from both methods underwent functional enrichment analysis via the STRING enrichment tool in Cytoscape software to identify significantly overrepresented KEGG pathways. Finally, overlapping hub proteins from both analyses were highlighted as the most promising candidates.

To determine potential candidate transcription factors involved in regulating candidate hub gene expression, a transcription factor enrichment analysis was conducted employing the ChIP-X Enrichment Analysis 3 (ChEA3) platform [26]. This analysis leveraged the ENCODE ChIP-Seq database [27] under default settings. The ChEA3 tool was employed to identify transcription factors linked to downstream differentially expressed genes by ranking transcription factors according to their relevance to the input gene sets. Specifically, the complete list of differentially expressed genes (FDR ≤ 0.10) was submitted to ChEA3, which interrogated the ENCODE ChIP-seq library comprising human, mouse, and rat ChIP-seq datasets. The core analysis of ChEA3 was performed utilizing default parameters, and transcription factors with an FDR ≤ 0.05 were deemed significantly enriched in relation to the differentially expressed gene sets.

The RNA-seq findings were verified through quantitative real-time polymerase chain reaction analysis of ten randomly chosen differentially expressed genes utilizing appropriate primers, in accordance with the previously described procedure [28]. Relative gene expression was determined utilizing the established method [29], with *β-actin* serving as the internal reference.

### 2.3. Statistical Analysis

The sample size was decided by utilizing the G*Power software (version 3.1; Heinrich-Heine-Universität Düsseldorf, Düsseldorf, Germany). Prior to analysis, data were tested for normality using the Shapiro–Wilk test and for homogeneity of variances using Levene’s test. Data analysis was performed utilizing one-way ANOVA in SAS software (version 9.4; SAS Institute Inc., Cary, NC, USA). To assess significant differences among the group means, the unpaired Student’s *t*-test was applied. A threshold of *p* < 0.05 was set to establish statistical significance. Specific statistical procedures related to the transcriptome analysis are mentioned in the respective methodological sections. Data visualization was conducted utilizing GraphPad Prism 8 (GraphPad Software Inc., San Diego, CA, USA).

## 3. Results

### 3.1. Identification of Differentially Expressed Genes

**Figure 1:**
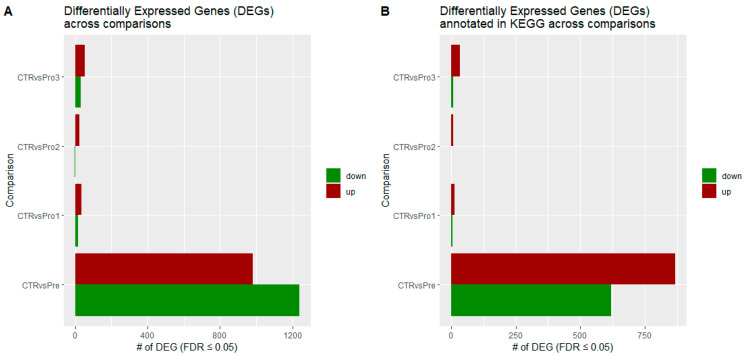
Bar charts showing (**A**) the number of upregulated and downregulated differentially expressed genes (DEGs) and (**B**) the number of DEGs annotated in the KEGG database across four comparison groups: control versus probiotic-3 (CTR vs. Pro3), control versus probiotic-2 (CTR vs. Pro2), control versus probiotic-1 (CTR vs. Pro1), and control versus prebiotic (CTR vs. Pre). Red bars indicate upregulated DEGs, while green bars indicate downregulated DEGs.

A total of 2221 genes were identified as differentially expressed between the control and prebiotic groups. In comparison, 48, 25, and 85 genes were differentially expressed between the control and probiotic-1, control and probiotic-2, and control and probiotic-3 groups, respectively (Figure 1A; FDR ≤ 0.05). Within the control versus prebiotic groups, 1434 of the 2221 differentially expressed genes were annotated for *Gallus gallus* in the KEGG database. Correspondingly, 18 out of 48 genes in control versus probiotic-1, 9 out of 25 in control versus probiotic-2, and 45 out of 85 in control versus probiotic-3 were annotated (Figure 1B; FDR ≤ 0.05).

**Figure 2:**
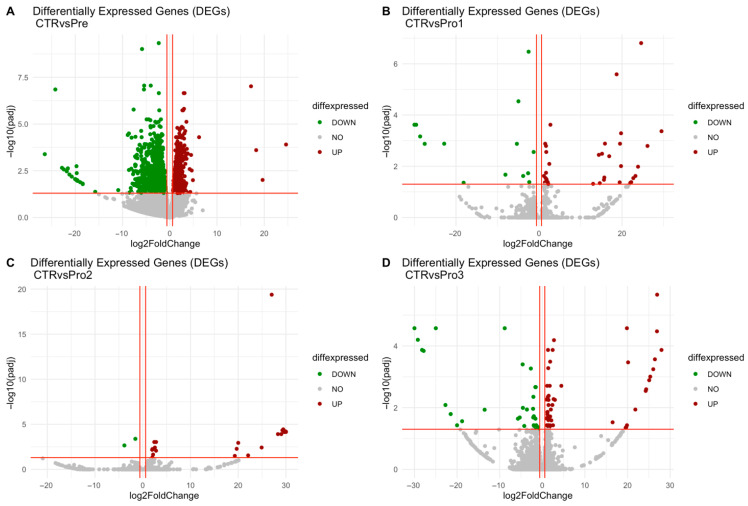
Volcano plots of differentially expressed genes (DEGs) for the following comparisons: control vs. prebiotic (CTR vs. Pre) (**A**), control vs. probiotic-1 (CTR vs. Pro1) (**B**), control vs. probiotic-2 (CTR vs. Pro2) (**C**), and control vs. probiotic-3 (CTR vs. Pro3) (**D**). The *x*-axis shows the log_2_ fold change, and the *y*-axis shows the −log_10_ adjusted *p*-value. Red and green dots indicate significantly upregulated and downregulated DEGs, respectively, while grey dots represent genes without significant differential expression.

In the comparison between control and prebiotic groups, 980 genes were upregulated, with log_2_ fold changes ranging from 0.69 to 24.62, while 1241 genes were downregulated, with log_2_ fold changes ranging from −0.74 to −26.46, as shown in Figure 2A. In the control versus probiotic-1 comparison, 34 genes were upregulated, with log_2_ fold changes ranging from 1.18 to 29.50, and 14 genes were downregulated, with values ranging from −1.29 to −30.00, as presented in Figure 2B. For the control versus probiotic-2 comparison, 23 genes were upregulated, with log_2_ fold changes between 1.97 and 30.00, whereas 2 genes were downregulated, with changes of −1.55 and −3.85, as illustrated in Figure 2C. In the control versus probiotic-3 comparison, 54 genes were upregulated, with log_2_ fold changes ranging from 0.94 to 27.98, and 31 genes were downregulated, with changes ranging from −1.00 to −30.00, as indicated in Figure 2D. The top five upregulated and downregulated KEGG-annotated differentially expressed genes for each comparison group are summarized in Table 1.

### 3.2. Pathway Enrichment Analysis of Differentially Expressed Genes

**Figure 3:**
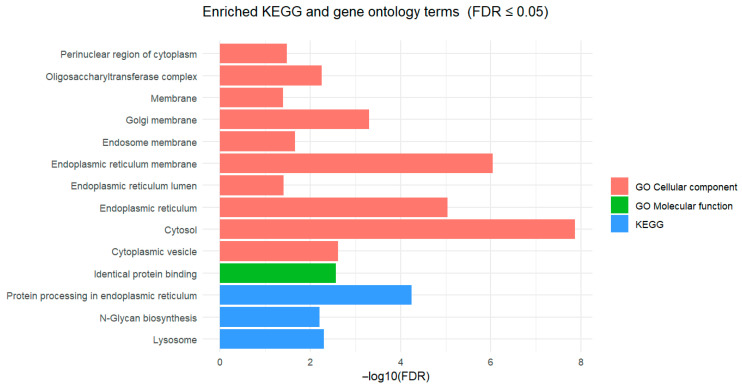
Pathway enrichment analysis of differentially expressed genes using Kyoto Encyclopedia of Genes and Genomes (KEGG) and Gene Ontology (GO) annotations. The *x*-axis shows the −log_10_ false discovery rate (FDR), reflecting enrichment significance, and the *y*-axis lists the significantly enriched KEGG pathways and GO terms. Red and green bars denote GO terms belonging to the cellular component and molecular function categories, respectively, while blue bars indicate enriched KEGG pathways.

Enrichment analyses using the Gene Ontology and KEGG databases via DAVID software identified significant results (FDR ≤ 0.05) exclusively for the control versus prebiotic and control versus probiotic-3 comparisons, as summarized in Table 2 and Figure 3. Specifically, for the control versus prebiotic comparison, the molecular function category of Gene Ontology was significantly enriched for identical protein binding. Concurrently, the cellular component category of Gene Ontology showed significant enrichment across multiple structures, including cytosol, endoplasmic reticulum membrane, endoplasmic reticulum, Golgi membrane, cytoplasmic vesicle, Oligosaccharyltransferase complex, endosome membrane, perinuclear region of the cytoplasm, endoplasmic reticulum lumen, and lysosomal membrane. In addition, KEGG pathway analysis revealed enrichment in protein processing in the endoplasmic reticulum, lysosome, and N-glycan biosynthesis pathways. In contrast, the control versus probiotic-3 comparison showed significant Gene Ontology enrichment for Phosphatidate phosphatase activity in the molecular function category and for the cytosol in the cellular component category.

**Figure 4:**
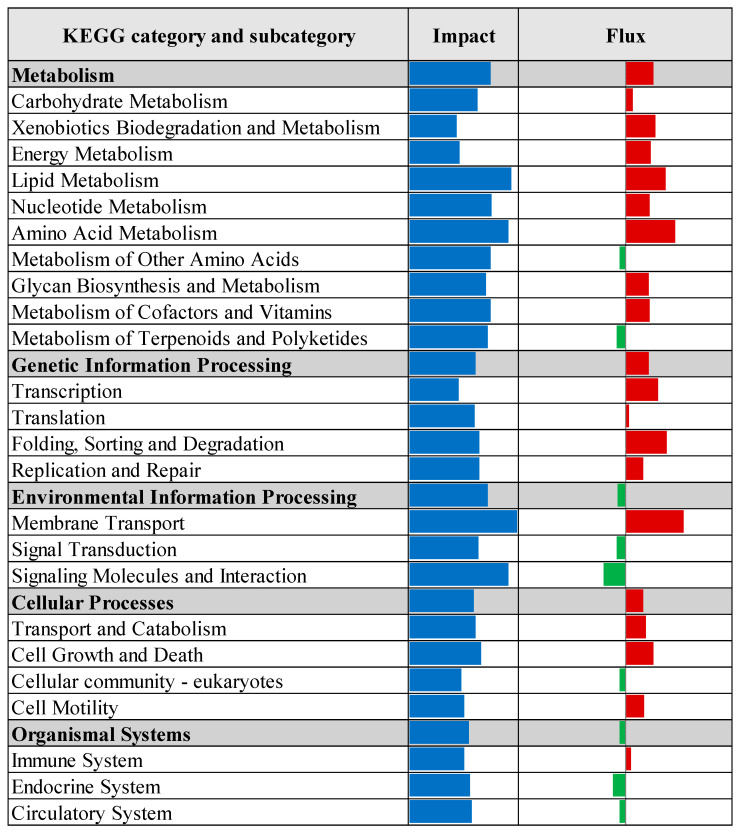
Summary of primary Kyoto Encyclopedia of Genes and Genomes (KEGG) categories identified by the Dynamic Impact Approach (DIA) analysis of differentially expressed genes in liver tissue of laying hens, comparing control and prebiotic-treated groups. The columns depict the overall impact and direction of flux. Flux values are color-coded: green bars indicate downregulated pathways (negative flux), and red bars indicate upregulated pathways (positive flux).

**Figure 5:**
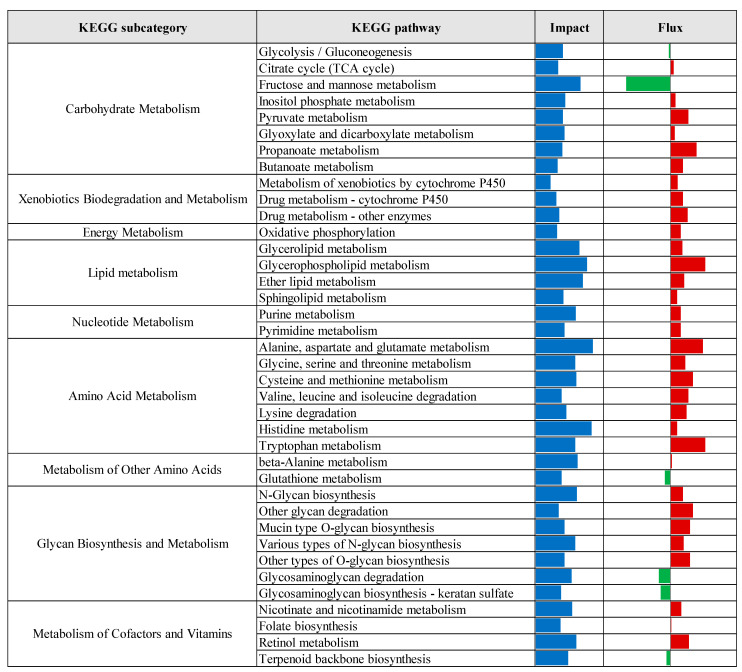
Summary of Kyoto Encyclopedia of Genes and Genomes (KEGG) metabolic pathways identified by the Dynamic Impact Approach (DIA) analysis of differentially expressed genes in liver tissue of laying hens, comparing control and prebiotic-treated groups. The columns show the overall impact and direction of flux. Flux values are color-coded: green bars indicate downregulated pathways (negative flux), and red bars indicate upregulated pathways (positive flux).

**Figure 6:**
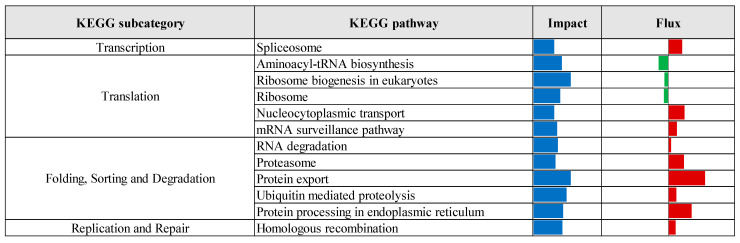
Summary of KEGG genetic information processing pathways identified through Dynamic Impact Approach analysis of differentially expressed genes in liver tissue of laying hens, comparing control and prebiotic-treated groups. The columns represent the overall impact and direction of flux. Flux values are indicated by color: green bars represent downregulated pathways (negative flux), and red bars represent upregulated pathways (positive flux).

**Figure 7:**
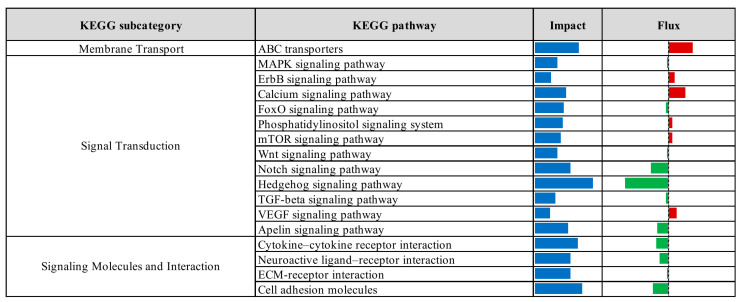
Summary of KEGG environmental information processing pathways identified by Dynamic Impact Approach analysis of differentially expressed genes in liver tissue of laying hens, comparing control and prebiotic-treated groups. The columns represent the overall impact and direction of flux. Flux values are indicated by color: green bars represent downregulated pathways (negative flux), and red bars represent upregulated pathways (positive flux).

**Figure 8:**
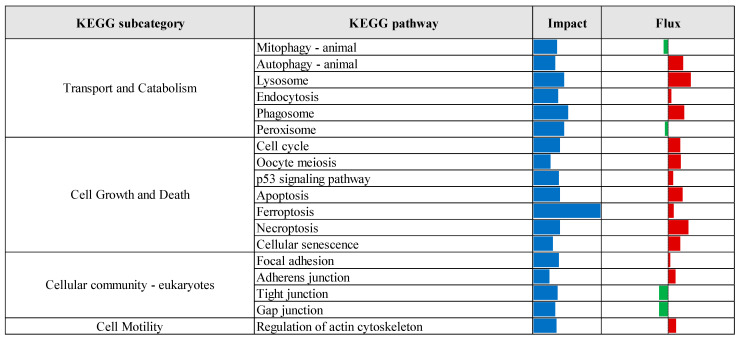
Summary of KEGG cellular processes pathways identified by Dynamic Impact Approach analysis of differentially expressed genes in liver tissue of laying hens, comparing control and prebiotic-treated groups. The columns represent the overall impact and direction of flux. Flux values are indicated by color: green bars represent downregulated pathways (negative flux), and red bars represent upregulated pathways (positive flux).

**Figure 9:**
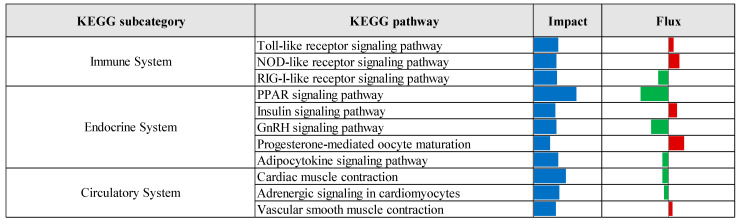
Summary of KEGG organismal systems pathways identified by Dynamic Impact Approach analysis of differentially expressed genes in liver tissue of laying hens, comparing control and prebiotic-treated groups. The columns represent the overall impact and direction of flux. Flux values are indicated by color: green bars represent downregulated pathways (negative flux), and red bars represent upregulated pathways (positive flux).

KEGG pathway enrichment analysis, based on the results of the Dynamic Impact Approach, revealed significant alterations exclusively in the control versus prebiotic comparison, as summarized in Figure 4. This analysis provides an overview of the overall impact and directional flux within major KEGG categories. Categories related to metabolism, genetic information processing, and cellular processes were predominantly upregulated, whereas those associated with environmental information processing and organismal systems were largely downregulated. Specifically, upregulation was observed across multiple KEGG subcategories, including carbohydrate metabolism, xenobiotics biodegradation and metabolism, energy metabolism, lipid metabolism, nucleotide metabolism, amino acid metabolism, glycan biosynthesis and metabolism, and metabolism of cofactors and vitamins. Additionally, processes related to transcription, translation, protein folding, sorting and degradation, as well as replication and repair, were upregulated. Cellular processes such as membrane transport, transport and catabolism, cell growth and death, cell motility, and immune system functions also exhibited upregulation. In contrast, downregulation was detected in subcategories associated with metabolism of other amino acids, metabolism of terpenoids and polyketides, signal transduction, signaling molecules and interaction, cellular community (eukaryotes), endocrine system, and circulatory system. Detailed pathway-level results within these KEGG subcategories are presented in Figure 5, Figure 6, Figure 7, Figure 8 and Figure 9.

**Figure 10:**
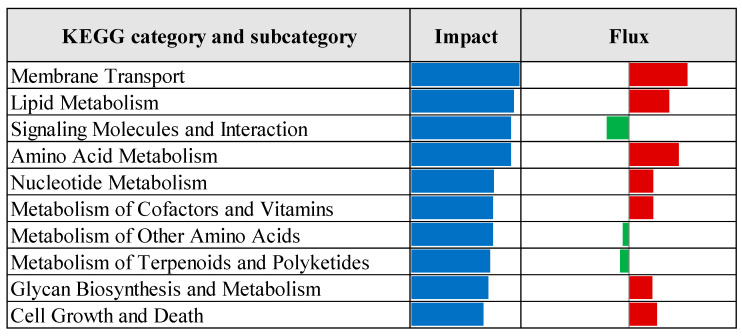
Top ten most impacted KEGG categories and subcategories identified by Dynamic Impact Approach analysis of differentially expressed genes in the liver tissue of laying hens, comparing control and prebiotic-treated groups. The columns represent the overall impact and direction of flux. Flux values are color-coded: green bars represent downregulated pathways (negative flux), and red bars represent upregulated pathways (positive flux).

The Dynamic Impact Approach analysis, as presented in Figure 10, revealed that the most impacted KEGG pathways in the prebiotic group compared to the control group included upregulation of pathways related to membrane transport, lipid metabolism, amino acid metabolism, nucleotide metabolism, metabolism of cofactors and vitamins, glycan biosynthesis and metabolism, and cell growth and death. Conversely, pathways associated with signaling molecules and interactions, metabolism of other amino acids, and metabolism of terpenoids and polyketides were downregulated in the prebiotic group relative to the control.

**Figure 11:**
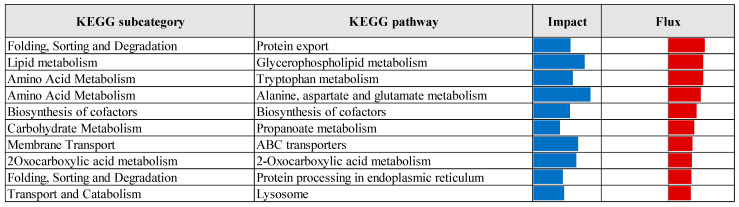
Top ten upregulated KEGG pathways identified by Dynamic Impact Approach analysis of differentially expressed genes in the liver tissue of laying hens, comparing prebiotic-treated and control groups. Columns represent the overall impact and direction of pathway flux. Positive flux values, indicating upregulation, are shown as red bars.

**Figure 12:**
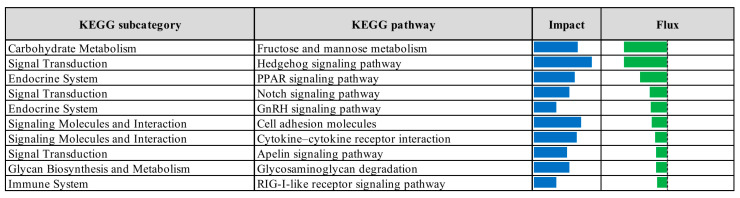
Top ten downregulated KEGG pathways identified by Dynamic Impact Approach analysis of differentially expressed genes in the liver tissue of laying hens, comparing prebiotic-treated and control groups. Columns represent the overall impact and direction of pathway flux. Negative flux values, indicating downregulation, are shown as green bars.

The top ten significantly upregulated KEGG pathways identified through Dynamic Impact Approach analysis of differentially expressed genes in the prebiotic group compared to the control group (Figure 11) included pathways related to protein export, glycerophospholipid metabolism, amino acid metabolism (including tryptophan, alanine, aspartate, and glutamate), cofactor biosynthesis, propanoate metabolism, ABC transporters, 2-oxocarboxylic acid metabolism, protein processing in the endoplasmic reticulum, and lysosomal function. In contrast, the top ten significantly downregulated pathways in the prebiotic group relative to control (Figure 12) encompassed fructose and mannose metabolism, hedgehog, peroxisome proliferator-activated receptors (PPARs), Notch, and gonadotropin-releasing hormone (GnRH) signaling pathways, as well as pathways associated with cell adhesion molecules, cytokine–cytokine receptor interactions, apelin signaling, glycosaminoglycan degradation, and RIG-I-like receptor signaling.

### 3.3. Construction of Protein–Protein Interaction Network and Screening of Hub Genes

**Figure 13:**
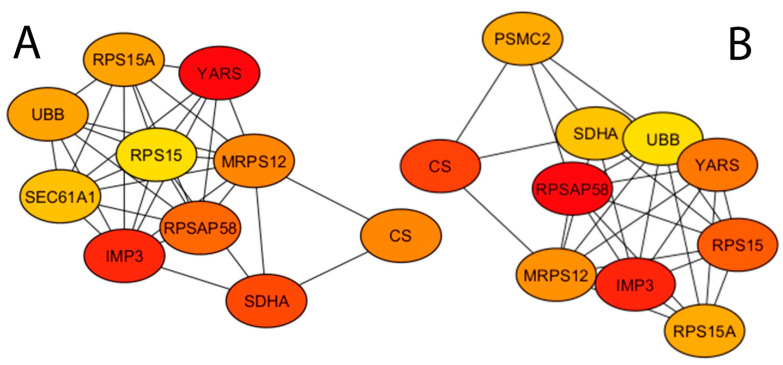
Hub genes identified within the protein–protein interaction network using the cytoHubba plugin in Cytoscape. Two ranking algorithms were applied: (**A**) Degree method and (**B**) Radiality method. The color gradient from red to yellow represents the ranking of hub genes, with red indicating higher-ranked nodes and yellow indicating lower-ranked ones, as reported in Table 3.

Using the list of differentially expressed genes at FDR ≤ 0.05, a protein–protein interaction network was constructed comprising 1474 nodes and 6911 edges. Hub proteins, characterized by multiple connecting edges, were identified within the network using global and local ranking methods (Degree and Radiality analyses) implemented in the cytoHubba tool (version 0.1). The top 10 identified hub nodes are presented in Table 3 and Figure 13. Notably, nine of these hub nodes were consistently identified by both methods and thus designated as significant hub proteins. These include UBB (ubiquitin B), CS (citrate synthase), SDHA (succinate dehydrogenase complex flavoprotein subunit A), MRPS12 (mitochondrial ribosomal protein S12), RPS15 (ribosomal protein S15), RPSAP58 (ribosomal protein SA pseudogene 58), IMP3 (IMP U3 small nucleolar ribonucleoprotein 3), YARS (tyrosyl-tRNA synthetase 1), and RPS15A (ribosomal protein S15a).

Subsequently, functional enrichment analysis using STRING was performed on these hub proteins to identify significantly overrepresented KEGG pathways. The analysis revealed significant enrichment of the Ribosome and Citrate cycle (TCA cycle) pathways (FDR ≤ 0.05; Table 4).

### 3.4. Detection of Transcription Factors

Transcription factor enrichment analysis using the ChEA3 tool (Chip-X Enrichment Analysis, Version 3), based on the ENCODE ChIP-seq database (https://www.encodeproject.org/chip-seq/transcription_factor/ (accessed on 17 August 2024)), identified 105 transcription factors significantly associated with the differentially expressed genes at FDR ≤ 0.05. The top five upstream regulators are listed in Table S2 and include MYOG (myogenin), CEBPB (CCAAT/enhancer-binding protein beta), USF1 and USF2 (upstream stimulatory factors 1 and 2), and JUND (junD proto-oncogene, a component of the AP-1 transcription factor complex).

## 4. Discussion

A substantial number of differentially expressed genes were identified exclusively in the control versus prebiotic comparison, enabling robust functional analyses; consequently, this discussion focuses on that comparison. A consistent regulatory pattern emerged across multiple KEGG terms, notably the pronounced downregulation of the fructose and mannose metabolism pathway within the carbohydrate metabolism subcategory. This finding is significant given that nondigestible carbohydrates, particularly fructose-based oligosaccharides (fructans), are recognized as key prebiotics [30]. Additionally, fructose plays a critical role in modulating intestinal barrier function and hepatic health [31]. The downregulation of this pathway was primarily driven by reduced expression of the KHK (ketohexokinase) gene, which encodes the enzyme responsible for the rate-limiting conversion of fructose to fructose-1-phosphate. In the liver, fructokinase rapidly phosphorylates fructose to fructose-1-phosphate, which is subsequently cleaved into dihydroxyacetone phosphate and glyceraldehyde by aldolase B [32]. Overall, the observed suppression of fructose and mannose metabolism supports the hypothesis that prebiotic supplementation may mitigate metabolic disturbances associated with high fructose intake [33]. Collectively, these results suggest that a prebiotic diet enhances intestinal fructose processing and protects the liver from excessive lipogenesis, without impairing overall carbohydrate and energy metabolism. This is supported by the slight upregulation of the broader carbohydrate metabolism category despite the marked downregulation of the fructose metabolism pathway, primarily driven by increased expression of genes involved in propanoate, butanoate, and pyruvate metabolism. Notably, inulin, a well-characterized prebiotic, has been reported to elevate intestinal SCFAs, including propionate and butyrate [34]. SCFAs such as acetate, propionate, butyrate, and lactate are predominantly produced through gut microbiota fermentation [35], and their positive effects on egg quality and safety in layer hens have been recently documented [36]. Specifically, SCFAs as microbial metabolites modulate calcium utilization and deposition via influencing systemic calcium homeostasis, thus enhancing eggshell quality. In addition, SCFAs lower intestinal pH, creating an unfavorable environment for pathogenic bacteria, thereby reducing the risk of microbial translocation into eggs [36]. These findings likely reflect prebiotic-induced enhancement of beneficial gut microbiota populations, consistent with a previous study showing increased abundance of *Lactobacillus* and *Olsenella* genera and elevated expression of microbial genes involved in propanoate and butanoate metabolism in prebiotic-fed layers [37]. In this context, it is important to emphasize that gut microbial communities produce a diverse array of metabolites involved in host physiological functions such as energy supply, intercellular communication, and immune regulation [38]. Notably, SCFAs and tryptophan catabolites have been implicated in modulating host–microbiota cross-talk [39]. The upregulation of genes such as LDHA (lactate dehydrogenase A), which catalyzes the reversible conversion of pyruvate to lactate, and ACSM5 (acyl-CoA synthetase medium-chain family member 5), responsible for activating fatty acids by conjugating them to CoA to form acyl-CoA, may contribute to this regulatory network. Furthermore, the marked downregulation of the HMGCS2 (3-hydroxy-3-methylglutaryl-CoA synthase 2) gene, which encodes a mitochondrial enzyme catalyzing the initial step of ketogenesis, a metabolic pathway that supplies lipid-derived energy during carbohydrate scarcity, indirectly indicates improved carbohydrate availability in prebiotic-supplemented chicks [40].

SCFAs have been reported to enhance intestinal nutrient utilization by stimulating the proliferation and differentiation of intestinal epithelial cells, leading to increased villus height and absorptive surface area [41]. This mechanism is particularly significant, as an expanded absorptive surface facilitates the digestion and absorption of nutrients, including monosaccharides and amino acids. Among the various effects, the SCFA-induced improvement in intestinal morphology may also promote calcium absorption, indirectly contributing to increased calcium deposition in eggshells and improved eggshell thickness, as recently demonstrated [42]. Of particular interest is the upregulation of ACAT2 (acetyl-CoA acetyltransferase 2) within the butanoate and pyruvate metabolic pathways. ACAT2, a key enzyme responsible for cholesterol esterification, is predominantly expressed in hepatocytes [43]. Cholesterol esterification facilitates cholesterol storage and transport while preventing cellular toxicity caused by excess free cholesterol [44]. Mechanistically, increased ACAT2 activity promotes the conversion of acetyl-CoA into acetoacetyl-CoA, thereby linking pyruvate and butanoate metabolism to lipid biosynthesis. This enhances the channeling of excess acetyl-CoA into cholesteryl ester formation, reducing free cholesterol burden while ensuring its safe packaging for lipoprotein assembly and export [43]. More broadly, the gut microbiota has recently emerged as a critical regulator of both cholesterol and bile acid metabolism [45]. Although no significant upregulation of bile acid metabolism was observed in this study, the increased expression of SCP2 (sterol carrier protein 2), an important gene involved in the primary bile biosynthesis pathway, supports a potential role for the microbiota in this process. SCP2 is known to promote the utilization of cholesterol in enzymatic reactions where cholesterol serves as the substrate [46]. Previous research has shown that prebiotic supplementation in broiler diets reduces serum cholesterol levels [47]. Considering that bile acid synthesis in the liver constitutes the primary route of cholesterol excretion [48], it is plausible that the gut microbiota influences bile acid metabolism. Regarding lipid metabolism, upregulation was observed in several pathways, including glycerophospholipid metabolism, glycerolipid metabolism, ether lipid metabolism, and sphingolipid metabolism. These alterations in lipid metabolism associated with prebiotic supplementation have been previously described in the literature [49]. Specifically, the observed upregulation of the glycerophospholipid metabolism pathway is consistent with recent findings in pigs, where gut microbiota significantly modulated hepatic glycerophospholipid metabolism [50]. Within this pathway, notable upregulation was detected for several key genes, including ETNPPL (ethanolamine-phosphate phospho-lyase), which catalyzes the pyridoxal-phosphate-dependent degradation of phosphoethanolamine into ammonia, inorganic phosphate, and acetaldehyde [51], as well as LPCAT3 (lysophosphatidylcholine acyltransferase 3), PLA2G15 (phospholipase A2 group XV), and LPIN1 (lipin-1). Functionally, ETNPPL upregulation suggests enhanced ethanolamine turnover and remodeling of membrane phospholipids, while LPCAT3 may increase incorporation of polyunsaturated fatty acids into phosphatidylcholines, impacting membrane fluidity and lipid signaling. Similarly, PLA2G15 upregulation can elevate lysophospholipid production, influencing inflammatory and signaling cascades, and LPIN1 plays a central role in triacylglycerol synthesis, thereby modulating hepatic lipid storage and energy homeostasis [52]. Together, these processes may promote shifts in hepatic lipid composition and availability, with implications for metabolic homeostasis and energy allocation. The upregulation of the glycerolipid metabolism pathway warrants particular attention, as recent research identified hepatic glycerolipid metabolism as a critical factor influencing egg-laying rates in chickens [53]. This finding aligns with the established role of triglycerides as major components of egg yolk precursors [54]. Within this pathway, the MOGAT1 (monoacylglycerol O-acyltransferase 1) gene was found to be upregulated. This gene encodes an enzyme responsible for catalyzing the synthesis of diacylglycerols, which are key precursors of physiologically important lipids such as triacylglycerols and phospholipids. In contrast, the AGPAT2 (1-acylglycerol-3-phosphate O-acyltransferase 2) gene was observed to be downregulated. This gene is known to be critically involved in the biosynthesis of triglycerides and phospholipids and has previously been identified as a hub gene in the glycerolipid metabolism pathway, influencing egg yolk precursor formation [53].

The observed upregulation of the membrane transport subcategory within the environmental information processing category, driven by the increased activity of the ABC transporter pathway, aligns with the broader context described above. This is consistent with the established role of ABC transporters in diverse physiological processes, including the export of cholesterol, bile salts, and metabolic end-products [55]. Additionally, the pronounced downregulation of the PPAR signaling pathway merits attention. Peroxisome proliferator-activated receptors (PPARs) are a family of ligand-activated transcription factors that regulate multiple metabolic processes, notably fatty acid metabolism [56]. Within this pathway, strong downregulation of the PLIN1 (perilipin-1) and PLTP (phospholipid transfer protein) genes has been detected.

The marked upregulation of protein export is noteworthy, given that the gut microbiota has been shown to modulate host amino acid metabolism [57] and to influence host protein/amino acid uptake, transport, and metabolism [58]. Protein export involves the active transport of proteins from the cytoplasm to the extracellular environment, predominantly via the Sec-dependent pathway, which translocates newly synthesized proteins across the cell membrane [59]. Significantly, several Sec-family genes, including SEC61A1, SEC11A, SEC11C, SEC63, and SEC62, were found to be upregulated. This upregulation aligns with the enrichment of the amino acid metabolism category and the protein processing in the endoplasmic reticulum pathway. Furthermore, protein–protein interaction analysis supports these findings, as the KEGG ribosomal pathway was significantly overrepresented among the top ten ranked hub proteins. The involvement of the endoplasmic reticulum is further substantiated by the results of the Gene Ontology enrichment analysis, which is noteworthy given the endoplasmic reticulum’s central role in liver pathophysiology [60]. The observed upregulation in the glycan biosynthesis and metabolism category aligns with this context, as N-glycan synthesis is initiated within the endoplasmic reticulum [61]. Within the broader upregulation of amino acid metabolism, significant augmentations were observed in the pathways associated with tryptophan and alanine, aspartate, and glutamate metabolism. Although studies on tryptophan requirements in laying hens are limited, its significance is underscored by its involvement in producing several key metabolites [62]. Regulation of tryptophan concentration is critical for maintaining systemic homeostasis, as it intersects with key pathways related to nutrient sensing, metabolic stress responses, and immune function [63]. Moreover, tryptophan serves as a precursor for the synthesis of neurotransmitters such as serotonin, as well as indoleamine 2,3-dioxygenase and quinolinic acid, thereby contributing to stress reduction and appetite regulation in animals [64]. Finally, tryptophan acts as a rate-limiting factor in protein synthesis due to its relatively low concentration compared to other amino acids, underscoring its critical role, as highlighted in a recent review on amino acid requirements for laying hens [62]. In this study, significant upregulation of genes involved in tryptophan metabolism was detected, including ACMSD (aminocarboxymuconate semialdehyde decarboxylase), KYNU (kynureninase), TDO2 (tryptophan 2,3-dioxygenase), MAOA (monoamine oxidase A), TPH1 (tryptophan hydroxylase 1), HAAO (3-hydroxyanthranilate 3,4-dioxygenase), and DHTKD1 (dehydrogenase E1 and transketolase domain containing 1). Among these, KYNU and ACMSD are identified as key enzymes of the kynurenine pathway, with ACMSD responsible for the catalytic breakdown of tryptophan into NAD+ [65]. The intestinal microbiota modulates the kynurenine pathway by regulating tryptophan absorption prior to hepatic metabolism, where most kynurenine synthesis occurs. Additionally, commensal bacteria in the intestinal lumen enzymatically convert dietary tryptophan into indole and related metabolites, channeling it toward the indole biosynthetic pathway [66]. Activation of tryptophan metabolism along the kynurenine pathway has been shown to mitigate hyperinflammation and promote long-term immune tolerance [67]. Furthermore, the upregulation of pathways involved in alanine, aspartate and glutamate, glycine, serine and threonine, and cysteine and methionine metabolism suggests enhanced antioxidant and anti-inflammatory potential in prebiotic-supplemented birds, consistent with the antioxidative properties of amino acids such as glycine, arginine, glutamine, methionine, cysteine, and tryptophan [68]. In this regard, the modest downregulation of the glutathione metabolism pathway, primarily due to reduced expression of GPX1 (glutathione peroxidase 1) and GSTM2 (glutathione S-transferase Mu 2), may reflect a reduction in oxidative stress.

The modest downregulation of the environmental information processing category, despite the pronounced upregulation observed in its membrane transport subcategory, is considered noteworthy. This pattern is primarily attributed to the significant downregulation of the hedgehog signaling and Notch signaling pathways within the broader category. Notably, activation of the hedgehog signaling pathway has been associated with hepatic accumulation of inflammatory cells [69]. Therefore, the downregulation of this pathway may be indicative of a reduced inflammatory state. Specifically, within the hedgehog pathway, DDH (desert hedgehog signaling molecule) and GLI-1 and GLI-2 (GLI family zinc finger protein 1 and 2) genes were found to be downregulated. Additionally, the slight downregulation of the TGF-beta and WNT signaling pathways aligns with this finding, as GLI family members are known to regulate transcription of pleiotropic TGF-beta target genes [70] and modulate the expression of factors involved in WNT signaling [71]. Regarding the impacts on immune function, a slight overall upregulation of the immune system KEGG subcategory was detected, primarily driven by the upregulation of Toll-like and NOD-like receptor signaling pathways, along with the downregulation of the RIG-I-like receptor signaling pathway. Although the Toll-like receptor signaling pathway was generally upregulated, significant gene-specific regulation was observed within this pathway. Specifically, TLR3 (toll-like receptor 3) and IFNAR-1 and -2 (interferon alpha and beta receptor subunits) were found to be upregulated, whereas SPP1 (osteopontin), CD40 (CD40 molecule), JUN (Jun proto-oncogene, AP-1 transcription factor subunit), and IRF-5 and -7 (interferon regulatory factors 5 and 7) were significantly downregulated. The downregulation of SPP1, a multifunctional protein known to promote inflammatory responses in liver diseases [72], aligns with the observed suppression of the hedgehog signaling pathway, consistent with the role of osteopontin as a downstream target of hedgehog pathway activation [73]. Similarly, reduced expression of JUN, a regulator of proliferation, differentiation, transformation, and apoptosis [74], and IRF5, a key mediator of liver macrophage activation [75], further supports a shift toward a more anti-inflammatory state. Furthermore, the downregulation of the cytokine–cytokine receptor interaction pathway corroborates this trend, primarily reflecting decreased activity of immune-related genes such as IL10RA (interleukin 10 receptor subunit alpha) and IL13RA2 (interleukin 13 receptor subunit alpha 2). In terms of liver physiological function, suppression of the hedgehog and Notch pathways likely reduces hepatocyte proliferation and ductular reaction, processes that are often enhanced during liver injury and fibrosis. This dampening effect may thus help maintain tissue integrity by limiting excessive regenerative or fibrogenic signaling [76]. The slight downregulation of TGF-beta and WNT signaling pathways further supports this interpretation, as both pathways normally promote fibrosis, epithelial-to-mesenchymal transition, and metabolic reprogramming of hepatocytes. Their reduced activity is therefore consistent with a protective effect against fibrogenesis [76]. Meanwhile, the downregulation of the RIG-I-like receptor signaling pathway may decrease antiviral and pro-inflammatory responses, thereby alleviating chronic immune activation in hepatic tissue [77]. Lastly, the downregulation of cytokine–cytokine receptor interaction reflects diminished intercellular immune communication, potentially lowering inflammatory recruitment and signaling cascades in the liver microenvironment [77]. Together, these effects point toward a functional outcome of reduced inflammation, lower fibrogenic drive, and a more balanced liver homeostasis. Collectively, these findings suggest that prebiotic supplementation may attenuate inflammatory signaling and response; however, the precise mechanisms remain to be fully elucidated. The study’s findings provide a solid molecular framework for understanding the effects of specific probiotics and prebiotics in poultry liver physiology. While generalization to other poultry breeds is plausible, species-specific validation is essential. Although direct extrapolation to humans is limited, the identified genes and pathways may inform future translational studies, especially regarding host–microbiota interactions, metabolic modulation, and immune function.

## 5. Conclusions

This study demonstrates that dietary supplementation with a yeast-derived prebiotic induces substantial transcriptomic alterations in the liver of laying hens, significantly exceeding those observed with the three different probiotic formulations. The prebiotic group demonstrated over 2200 differentially expressed genes compared to the control, with enriched pathways linked to protein processing, lysosomal function, glycan biosynthesis, and a wide array of metabolic and cellular processes. Notably, KEGG-based Dynamic Impact Analysis revealed widespread upregulation in metabolic, genetic information processing, and cellular process pathways, while signaling-related and organismal systems pathways were predominantly downregulated. Protein–protein interaction analysis further identified key hub genes involved in ribosomal activity and the tricarboxylic acid cycle, suggesting enhanced protein synthesis and energy metabolism under prebiotic influence. Furthermore, transcription factor analysis implicated regulatory elements such as MYOG, CEBPB, and USF family members in orchestrating the observed gene expression patterns. These findings highlight the pronounced molecular effects of prebiotics on hepatic gene expression and metabolic pathways, supporting their role as potent modulators of host physiology in poultry production systems. Future studies should address the study’s limitations by validating the physiological relevance and long-term benefits of the observed molecular changes.

## Figures and Tables

**Table 1:** Top five upregulated and downregulated KEGG-annotated differentially expressed genes across comparison groups.

Rank	Expression	CTR vs. Pre ^1^		CTR vs. Pro1		CTR vs. Pro2		CTR vs. Pro3	
		Gene	log_2_FC ^2^	Gene	log_2_FC	Gene	log_2_FC	Gene	log_2_FC
1	upregulated	*ETNPPL*	6.2	*LECT2*	6.18	*ABCG8*	2.79	*NHLH1*	19.87
2		*CCNH*	4.65	*CKMT2*	4.38	*PPAT*	2.54	*KLHL30*	19.68
3		*MRPS18C*	4.22	*FKBP5*	7.14	*GPT2*	2.41	*CLCA1*	19.56
4		*MOGAT1*	3.59	*LPIN1*	4.14	*FKBP5*	2.41	*LPIN1*	2.74
5		*ACMSD*	3.56	*HSPH1*	3.99	*PAICS*	2.16	*ABHD3*	2.65
1	downregulated	*LRRC4C*	−8.63	*ELF3*	−5.33	*PAFAH2*	−1.55	*HEYL*	−4.61
2		*DHH*	−7.3	*KANK4*	−2.63	-	-	*ELF3*	−4.55
3		*SOX17*	−6.3	*PDE4D*	−2.51	-	-	*KIF21B*	−1.95
4		*CARNS1*	−6.16	*TRPM1*	−2.34	-	-	*CYP4A22*	−1.83
5		*DTX1*	−5.97	*ENPP2*	−1.29	-	-	*ARHGAP45*	−1.63

^1^ CTR vs. Pre, control versus prebiotic; CTR vs. Pro1, control versus probiotic-1; CTR vs. Pro2, control versus probiotic-2; CTR vs. Pro3, control versus probiotic-3. ^2^ log_2_FC, log_2_ fold change.

**Table 2:** Enriched Kyoto Encyclopedia of Genes and Genomes (KEGG) and Gene Ontology (GO) terms for differentially expressed genes.

Comparison ^1^	Database ^2^	Pathway/Term	*p*-Value	Fold Enrichment	FDR ^3^
CTR vs. Pre	KEGG	Protein processing in endoplasmic reticulum	3.70 × 10^−7^	2.24	5.77 × 10^−5^
		Lysosome	6.48 × 10^−5^	2.09	5.05 × 10^−3^
		N-Glycan biosynthesis	1.19 × 10^−4^	2.86	6.17 × 10^−3^
	GO-MF	Identical protein binding	2.37 × 10^−6^	1.55	2.73 × 10^−3^
	GO-CC	Cytosol	1.98 × 10^−11^	1.41	1.38 × 10^−8^
		Endoplasmic reticulum membrane	2.57 × 10^−9^	2.06	8.95 × 10^−7^
		Endoplasmic reticulum	3.96 × 10^−8^	1.78	9.20 × 10^−6^
		Golgi membrane	2.86 × 10^−6^	2.10	4.97 × 10^−4^
		Cytoplasmic vesicle	1.75 × 10^−5^	2.28	2.44 × 10^−3^
		Oligosaccharyltransferase complex	4.81 × 10^−5^	8.16	5.58 × 10^−3^
		Endosome membrane	2.17 × 10^−4^	2.62	2.16 × 10^−2^
		Perinuclear region of cytoplasm	3.82 × 10^−4^	1.65	3.32 × 10^−2^
		Endoplasmic reticulum lumen	5.00 × 10^−4^	3.00	3.87 × 10^−2^
		Lysosomal membrane	5.79 × 10^−4^	1.41	4.03 × 10^−2^
CTR vs. Pro3	GO-MF	Phosphatidate phosphatase activity	4.72 × 10^−4^	2.65	2.83 × 10^−2^
	GO-CC	Cytosol	4.93 × 10^−4^	8.70 × 10^1^	5.03 × 10^−2^

^1^ CTR vs. Pre, control versus prebiotic; CTR vs. Pro3, control versus probiotic-3. ^2^ GO-MF, GO-molecular function; GO-CC, GO-cellular component. ^3^ FDR, false discovery rate.

**Table 3:** Top 10 ranked hub proteins identified from the protein–protein interaction network using the cytoHubba plugin in Cytoscape. Hub genes were determined by applying two algorithms: Degree and Radiality methods.

Rank	Hub by Degree Method ^1^	Hub by Radiality Method
1	YARS	RPSAP58
2	IMP3	IMP3
3	SDHA	CS
4	RPSAP58	RPS15
5	MRPS12	YARS
6	CS	MRPS12
7	UBB	RPS15A
8	RPS15A	PSMC2
9	SEC61A1	SDHA
10	RPS15	UBB

^1^ YARS, tyrosyl-tRNA synthetase; IMP3, IMP U3 small nucleolar ribonucleoprotein 3; SDHA, succinate dehydrogenase complex flavoprotein subunit A; RPSAP58, ribosomal protein SA pseudogene 58; MRPS12, mitochondrial ribosomal protein S12; CS, citrate synthase; UBB, ubiquitin B; RPS15A, ribosomal protein S15a; SEC61A1, Sec61 translocon alpha subunit 1; RPS15, ribosomal protein S15; PSMC2, proteasome 26S subunit ATPase 2.

**Table 4:** The most enriched KEGG pathways identified by STRING enrichment analysis based on the top 10 hub proteins from the protein–protein interaction network, determined utilizing both the Degree and Radiality methods.

KEGG Pathway	Number of Genes	*p*-Value	FDR	Genes ^1^
Ribosome	109	3.96 × 10^−5^	0.00658	*MRPS12*, *RPS15*, *RPSAP58*, *RPS15A*
Citrate cycle (TCA cycle)	27	0.0107	0.024	*SDHA*, *CS*

^1^ MRPS12, Mitochondrial ribosomal protein S12; RPS15, Ribosomal protein S15; RPSAP58, Ribosomal protein SA pseudogene 58; RPS15A, Ribosomal protein S15a; SDHA, Succinate dehydrogenase complex flavoprotein subunit A; CS, Citrate synthase.

## Data Availability

The data are available upon reasonable request from the corresponding authors.

## Supplementary Materials

The following Supporting Information can be downloaded at: https://www.mdpi.com/article/10.3390/vetsci12090822/s1, Table S1: Nutrient composition of the control diet (as-fed basis); Table S2: Top five enriched transcription factors (TF) identified by ChEA3 analysis using the ENCODE ChIP-seq database.
