# Supplementary material for: RNA Sequencing-Based Transcriptome Analysis of Liver in Laying Hens Supplemented with Dietary Probiotic Bacillus Species and Prebiotic Yeast (Saccharomyces cerevisiae) Cell Walls
Source: Vet Sci. 2025 Aug 27;12(9):822. doi: 10.3390/vetsci12090822 (PMC12474164; doi:10.3390/vetsci12090822)
Supplement: Supplementary file 1 [file vetsci-12-00822-s001.zip › vetsci-3825129-supplementary.pdf]

Table S1: Nutrient composition of the control diet (as-fed basis).

Constituents	Quantity, g/kg	Nutritional composition	Quantities
Corn	556	Metabolizable energy, MJ/kg	11.5
Soybean meal	276	Crude protein, g/kg	170
Wheat bran	25.0	Non-phytate phosphorus, g/kg	4.6
Corn oil	20.0	Calcium, g/kg	40
Di-calcium phosphate	16.0	Lysine, g/kg	7.6
Limestone	100	Total sulfur amino acids, g/kg	6.8
Sodium chloride	3.00	Threonine, g/kg	5.8
DL-methionine	2.00		
Vitamin–mineral mix [1]	2.00		

[1] Supplement provided the following per kilogram of diet: vitamin A (trans-retinyl acetate), 6600 IU; vitamin D3 (cholecalciferol), 2695 IU; vitamin E (all-rac-tocopherol acetate), 15 mg; vitamin K (bisulfate menadione complex), 1.2 mg; riboflavin, 4.4 mg; pantothenic acid (d-calcium pantothenate), 6.6 mg; niacin 21 mg; choline (choline chloride), 358 mg; vitamin B12 (cyanocobalamin), 0.006 mg; manganese ($MnSO_4 \cdot H_2O$), 83 mg; zinc (ZnO), 61 mg; iron ($FeSO_4 \cdot H_2O$), 32 mg; copper ($CuSO_4 \cdot 5H_2O$), 3.9 mg; iodine (KI), 1.1 mg; selenium (Na_2SeO_3), 0.256 mg.

Table S2: Top five enriched transcription factors (TF) identified by ChEA3 analysis using the ENCODE ChIP-seq database.

TF[1]	Intersect	FET *p*-value[2]	FDR[3]	Overlapping Genes
MYOG	392	1.35E-17	7.40E-18	*ATF1, GHITM, RBPJ, AKT1, DICER1, FRS3, HSPG2, PRKAR1A, UNC50, CDIP1, TBPL1, MTMR1, MEAF6, SAR1B, AGAP1, GATA3, PCNX1, RDH10, ATG7, LNPEP, EIF2AK4, ARF4, SNX11, CTBS, PCMTD1, LYPLA1, PGAM1, F2, RBSN, SIRT1, FAHD2A, MRPL43, F5, MED21, ERGIC3, CACUL1, SPOP, PMS1, CRBN, MDH1, STAT1, SAMD4A, SNX21, PLXDC1, MITD1, EXOC3L1, CSE1L, UBE2D3, PLOD2, OLFML2A, KPNA4, KPNA1, SH3GLB1, BNIP3L, FRMD6, IL6ST, PLA2R1, RNASEH2B, ARL1, SLC9A1, AMDHD1, BRIX1, FBXO33, FBXO31, NACA, CLPTM1L, ITGB1, DRAXIN, COX4I1, ARL5B, PGLS, SNAP29, ACTR3, SMIM15, VPS13A, ARNT, RABGGTB, ATRN, NPC1, ITGA6, BECN1, HSD17B10, BEND3, UBQLN1, CNNM4, NDUFA4, MID1IP1, TM6SF1, CABLES2, GSTZ1, EIF3G, PDCD4, EIF3E, EIF4A2, ERRFI1, PLEKHB1, ZDHHC6, CCNC, PPAT, TBC1D22A, SEC63, PAQR7, MOGS, KDELR2, GSPT2, MICAL1, SARAF, INTS7, PTPRU, CSRNP2, HIBADH, PTPRK, PCM1, AKAP11, SPTLC1, NUDCD1, SEC13, CACYBP, CD47, CRELD2, NAPA, DERL1, CRIP2, PTPA, PGRMC2, FAM172A, MON2, PNPLA8, MIB2, KLHL2, ORMDL1, DOHH, PEX13, PER2, VMP1, NEDD1, CCNYL1, TACC1, ESYT1, ETS1, NARS, CCND3, KIF5B, PALM, DNAJB12, SDHA, TANC2, PLA2G15, DENND6A, PSMD12, UBIAD1, NDUFB10, YTHDC2, SEL1L, HTRA2, CNN2, PACSIN2, RHPN1, KIF3C, OST4, UBE2B, VTA1, DEGS1, YWHAE, DDX3X, CHD9, YWHAB, ARHGEF10L, TNFAIP1, SEC61A1, MYBL2, UTP15, SUN1, DCAKD, PEX2, GPCPD1, SYNGR3, ELF1, GORASP1, NDUFS3, PTGES3L, IFT46, RRN3, ASAP3, AGPAT2, GK5, TNKS2, CNR1, SMNDC1, CYTH1, ARFGEF2, TTC13, LAMB3, GALNT2, ATP2B1, U2SURP, CLCN7, CCS, GCSH, NUB1, TFG, DYNC1LI1, NUP205, HIF1A, LDHA, NUP85, ST3GAL2, SNAPIN, IRF2BP2, TOLLIP, SMG1, BTG1, GMEB2, CLTB, MCEE, TRIM3, ANXA6, AP3S2, BRD1, TCF12, AFTPH, TUBB2B, ANGPTL4, PRUNE1, HSPA13, TM9SF3, TMEM168, SLC17A5, DEDD, DTD2, WWP2, HIPK3, ERP44, NMNAT1, LPCAT3, CYP20A1, LAMTOR2, GPR146, TUSC3, BZW2, PPP1CB, APMAP, SLC39A9, RNF44, GPT2, TPM2, CEP131, CSGALNACT2, CACNB1, RAB31, STIM2, ANKRD40, FKBP5, CUL5, INSIG1,*

				CUL2, ABHD8, ABHD6, PLXNA2, IGFBP7, TPPP, CD164, SEC16B, OSBPL7, WFS1, BTBD9, COPS4, SYNJ1, LRFN1, TADA3, PLEKHM1, CNOT8, COPS8, RNF13, PIK3C2A, NOB1, OPA1, AP1G1, WDR6, LBR, PATL1, C1QL1, DUSP23, WBP1, HECTD1, POLR2E, DNAJB9, MAL2, BAK1, UGGT1, SEC11C, SEMA4D, SEMA4B, CDC42BPB, VAPA, PSAT1, DNAJA2, TRAF3IP1, PIT-PNB, SLC35B1, TIAL1, RPS6KA3, GLUL, HABP2, PDK1, COG6, SACM1L, RUNX2, RUNX1, GSAP, STXBP1, CBARP, SCFD1, TRPM7, STAM, SMU1, TMCC2, CORO7, SLC22A15, SH3PXD2A, FFAR4, TMED7, MANBA, PARP4, AMPD2, RHOF, TYK2, RHOC, UCHL5, NFX1, MAPRE3, ZDHHC21, PURA, ERBIN, NR6A1, XPOT, MARS, RAB3GAP2, NDUFA10, MANEA, ARHGAP35, TATDN1, NSF, PDIA3, ALG12, PPP4R3A, PDIA5, PDIA4, CMPK1, SELENOW, STAM2, ABCG2, FBLN1, ADIPOR1, TMEM123, UBB, MAN2A1, GAS2L1, CBX6, PNPT1, CBX4, CPSF2, TBCD, DNAJC13, TBCK, MAN2B2, LRP12, USP36, ELAVL1, GMPPB, PPP2R5A, ABCC9, LATS1, VAC14, USP15, HLF, KDM3B, HSPD1, RMND5A, ARID3A, NAP1L4, DES, RPS6KB1, PITHD1, KANSL3, XPNPEP3, ATP13A2, ATP13A3, SSBP1, ABCE1, LPIN1, PAXBP1
CEBPB	380	5.90E-16	1.60E-13	ATF1, SLC4A1AP, GHITM, RBPJ, XYLB, SLC5A6, MYL6, MTHFD1, UNC50, SAR1B, AGAP1, MRPL17, C5, HLCS, AFMID, ATG7, EIF2AK3, BTBD11, CP, CS, ARF4, SNX13, SHF, SHE, HEY2, LYPLA2, LYPLA1, TMC7, NIPA2, F2, SIRT1, F5, F7, BDH2, TMEM33, TMEM106B, GPRC5C, OAT, ATL2, ZNF518A, SNX33, PSMB3, SPOP, SPP1, PMS1, CRBN, MDH1, STAT1, SNX21, RABIF, SNX17, MITD1, LGR4, TMEM167A, NAB2, CASC3, IL1RAP, LAPTM4A, SESN2, KPNA4, KPNA3, KPNA1, PIPOX, CPB2, UQCRB, UBFD1, SLC9A1, STK35, LRRC4C, RBPMS, FBXO33, FBXO31, SP2, SP5, TMEM19, NFE2L2, NADK2, ARL5B, SPR, PPP6R3, ITGAV, PGLS, BCAS2, MBL2, ACTR3, ACTR2, VPS37C, ARNT, ATRN, ARL4A, PPM1B, NPC1, ZNF638, AGXT, SEC23A, NDUFB6, SRC, NDUFB2, UBQLN1, RILP, SH2B3, SEC24A, MID1IP1, NICN1, TTC39B, EIF3M, CCM2, ERRFI1, TMEM41B, ECI1, TXNDC12, IKZF5, EFR3A, CDC27, SEC62, PAQR7, IMP3, ECH1, MOGS, BTD, PPA2, SOAT1, ALPK1, KCTD16, ZMPSTE24, APOH, ARSK, DHX36, APOB, PCK2, PAF1, INTS11, MRPS17, CSRNP2, PTPRK, SLC7A11, SPTLC1, NUDCD1, HYAL3, AASS, CCT2, SEC13, NCOA4, CACYBP, CANX, NAPA, CREG1, MON2, ORMDL1, DOHH, PEX13, PER2, VMP1, TFAP4, FOXA2, PNPLA2, KLB, NARS, SLC30A5, DNAJB12, MAT1A, PLA2G15, ADAM17, PSMD12, PSMD14, YTHDC2, PSEN1, PDS5A, ISOC1, RHPN1, YARS, UBE2B, NUP153, APPBP2, GNB4, DEGS1, STT3B, MXD1, YWHAB, TNFAIP1, TMEM222, IFT57, DDX59, NCBP1, DCAKD, ATP6AP2, GPCPD1, TTI2, GORASP2, ATG4B, IRF9, RRN3, DCTN2, SPOCK2, U2SURP, CLCN7, ERCC5, VPS29, IPO11, ABAT, RNF114, MPV17, RPL21, DYNC1LI1, BIN3, RPN2, RPN1, LDHA, PRDX4, RRS1, NRBF2, NHEJ1, JUND, NFATC3, NR1D1, PAFAH2, BTG1, TINAGL1, WDR48, ARPC1B, SERPINA10, CLTB, AHSG, TCF12, TIMM21, TMEM175, FAM210A, JDP2, ZNF593, TMEM168, RPS15A, RNF139, SLC17A5, PCGF1, AGA, F10, SERPIND1, DEF6, HIPK3, AGBL5, RNF149, RLIM, VKORC1L1, CSF3R, PROS1, LCLAT1, PPP1CB, ABHD13, IPO8, RNF44, IGFBP4, GPT2, USP3, ANKRD40, IFNAR1, ABHD3, CUL2, ABHD8, UBR2, KNG1, DDRGK1, PLXNA2, AP1M1, CD164, OSBPL6, PHKB, CNIH1, CNOT1, MAFF, SERINC3, UFSP2, OPA1, AP1G1, WDR6, SLC37A4, GPX1, DNMT3A, HAUS6, PLRG1, PATL1, PTP4A1, DUSP23, CEBPB, VPS4B, HECTD1, POLR2E, MAL2, SNRPB2, ASCC3, BAK1, UGGT1, CFLAR, PSAT1, PANK1, AQP9, SOX4, PDK1, COG6, SACM1L, AP1B1, WDPCP, TLN1, MGST1, LIAS, INPP5B, STRIP1, STC2, TRPM7, ARGLU1, CDT1, SMU1, HAL, ANP32E, HBS1L, FFAR4, TMED2, TMED7, MANBA, PARP4, SCNM1, NFX1, CLDN12, ALDOC, HAAO, METTL9, CYP2W1, PDP2, XPOT, MARS, NDUFA10, SPPL2A, TMEM14A, ALG12, ALG10, TMEM131, PDIA4, ILVBL, COPB1, DNAJC22, ADIPOR1, DTX4, SDCBP, UBB, DNAJC19, PNPT1, CBX4, CBX1, DNAJC11, TBCD, TBCC, TDRD7, SMAD6, CRLS1, COQ5, NFKBIB, USP36, ASNSD1, MFSD9, CYP4V2, NTMT1, DBT, FLOT2, POLG, FGA, FGG, ARAP3,

				PPP2R5A, LATS1, KDM3A, HLF, ETFDH, MOSPD2, ATP1A1, HSPD1, UGP2, ABCF3, GDAP2, XPNPEP3, SSBP1, LPIN1
USF1	222	1.42E-15	2.60E-13	*ATF1, TMEM97, SLC5A6, MTHFD1, PRKAR1A, CDIP1, TBPL1, HSPH1, PRKAR2B, FUCA2, ARF4, JPH1, SIRT1, MRPL43, BDH2, TMEM33, TMEM106B, CACUL1, TCOF1, PSMB3, SPP1, MTCH1, NAB2, LAPTM4A, OSBP, BNIP3L, CNPY3, RNASEH2B, ARL1, UBFD1, SBNO1, FBXO33, OSBPL1A, TMEM19, ARL5B, SNAP29, BCAS2, VPS37C, MIEN1, LRRC8D, EIF4E, SEC24A, TTC39B, CCM2, EIF3F, SLC25A36, CCNH, TXNDC16, CCDC91, PPAT, SEC62, SLC12A7, IMP4, EIF1B, PAICS, ALDH6A1, INTS6, FBXL5, KHK, NUDCD3, PCM1, AKAP11, SDF4, QSOX2, CANX, NAPA, DERL1, DERL2, PTPA, PGRMC2, PNPLA7, DOHH, TFAP4, CCNYL1, ESYT1, DPAGT1, SCARB2, KIF5B, BAHCC1, SLC30A7, SLC30A5, ADAM10, TANC2, DENND6A, UBE2B, IGF2R, CLK2, DEGS1, STT3B, DDX3X, YWHAB, EEF2K, RBM15, TTI2, GORASP1, CRY1, IRF9, RRN3, DCTN2, CLN5, CYTH3, CNR1, SMNDC1, ARFGEF2, TTC13, ATP2B1, U2SURP, TTC17, CLCN7, GLB1, WIPF2, SMC3, GCSH, STMN1, ADNP2, VPS35, MPV17, IER5, PDXDC1, GNS, HIF1A, LDHA, PRDX4, RRS1, ST3GAL5, NRBF2, NFATC3, NR1D1, ADK, ANXA6, GRAMD4, SLC17A5, KIDINS220, WWP2, LRPPRC, CYP20A1, RLIM, LAMTOR2, GPR146, CSF1, FMR1, LAMP1, RAB31, FKBP5, ANKRD16, CUL5, INSIG1, PSAP, UBR5, PLTP, CD164, TMEM132A, M6PR, TRAPPC8, SYNJ1, CNOT1, REEP3, MAFF, TPP2, AP1G1, GPX1, HAUS6, PATL1, PTP4A1, VPS50, DNAJB9, SEC11C, CFLAR, VAPA, VPS41, SLC35B1, RPS6KA5, MCCC1, RUNX3, MRPS18C, RUNX1, VDAC1, CBARP, STC2, TRPM7, STAM, RPS28, CORO7, TMED2, AMPD2, NFX1, FAM20C, ATP6V0C, NOL8, NR6A1, PDP2, XPOT, RAB3GAP2, ARHGAP35, TMEM131, ILVBL, SELENOW, MATR3, CBX6, EPHX1, DNAJC13, SMAD6, COQ4, NFKBIB, USP36, USP38, HS2ST1, MFSD5, POLG, URB1, VAC14, KDM3A, USP15, KDM3B, ETFDH, MOSPD1, SELENOF, MAPK3, KANSL1, XPNPEP3, ATP13A3, SSBP4*
USF2	229	6.02E-15	6.70E-13	*ATF1, TMEM97, SCP2, XYLB, SLC5A6, MTHFD1, CDIP1, PRKAR2B, FUCA2, NMT2, SNX13, SHF, LYPLA1, SIRT1, F5, CACUL1, RAPGEF3, ATL2, PSMB3, BID, MTCH1, CASC3, LAPTM4A, BNIP3L, CNPY3, RNASEH2B, FOXO6, UBFD1, SBNO1, FBXO33, OSBPL1A, TMEM19, MCFD2, ARL5B, SNAP29, BCAS2, VPS37C, NDUFB2, MIEN1, EIF4E, SEC24A, CCM2, EIF3F, SLC25A36, CCNH, ECI1, PLEKHB1, NUDT5, PPAT, SEC62, SLC12A7, IMP4, MOGS, BCORL1, EIF1B, PAICS, ALDH6A1, INTS6, FBXL5, INTS11, KHK, NUDCD3, PCM1, AKAP11, SDF4, QSOX2, NR5A2, CANX, NAPA, DERL1, DERL2, PTPA, PGRMC2, CREG1, PNPLA7, DOHH, TFAP4, CCNYL1, DPAGT1, SCARB2, KIF5B, SLC30A7, SLC30A5, ADAM10, MID2, DENND6A, PSEN2, PSEN1, UBE2B, IGF2R, CLK2, GNB4, STT3B, DDX3X, YWHAB, RBM15, ATP6AP2, TAPBP, GORASP1, PEX6, NDUFS3, IRF9, RRN3, DCTN2, CLN5, CYTH3, SMNDC1, ARFGEF2, ATP2B1, U2SURP, CLCN7, GLB1, CD7, DQX1, WIPF2, SMC3, GCSH, STMN1, ADNP2, VPS35, MPV17, IER5, LRPAP1, PDXDC1, GNS, HIF1A, LDHA, PRDX4, SSR1, ST3GAL5, NRBF2, NHEJ1, NFATC3, NR1D1, SMG1, ANXA6, SLC17A5, KIDINS220, NR1H4, WWP2, LRPPRC, CYP20A1, GPR146, FMR1, LAMP1, LAMP2, SEMA6B, CSGALNACT2, TAP1, RAB31, FKBP5, ANKRD16, CUL5, GLO1, INSIG1, PSAP, PLTP, CD164, TRAPPC8, SYNJ1, TPP2, AP1G1, GPX1, TRAF2, PATL1, DNAJC3, HDAC7, VPS50, DNAJB9, SEC11C, SEMA4B, CUL4A, VAPA, VPS41, SLC35B1, IL1R2, MCCC1, RUNX3, MRPS18C, RUNX1, STXBP1, CBARP, STC2, TRPM7, RPS28, CORO7, TMED2, CBFA2T3, NFX1, ATP6V0C, NOL8, MAP4K3, ERBIN, BATF, NR6A1, PDP2, XPOT, RAB3GAP2, ARHGAP35, TMEM131, ARHGAP45, ILVBL, SELENOW, MATR3, ABCG4, ADIPOR1, CBX6, DNAJC12, DNAJC13, SMAD6, DNAJC18, COQ4, LRP12, NFKBIB, USP36, USP38, DHTKD1, HS2ST1, MFSD5, NTMT1, VAC14, KDM3A, MOSPD2, MOSPD1, SELENOF, MAPK3, KANSL1, XPNPEP3, ATP13A3*
JUND	329	4.04E-14	3.70E-12	*RBPJ, XYLB, GSTK1, FRS3, CD2AP, SLC5A6, MYL6, PSME4, UNC50, SAR1B, MRPL17, AAAS, HSPH1, AFMID, JUN, FUCA2, MRPL24, EIF6, SHF, TMC7,*

NIPA2, F2, FAHD2A, F5, F7, TMEM33, AGPS, TMEM106B, GPRC5C, RAPGEF3, ATL2, SRPX2, SPOP, CRBN, STAT1, RABIF, SNX17, MTCH1, CSE1L, UBE2D3, LAPTM4A, OSBP, KPNA3, BNIP3L, TPST1, UQCRB, UBFD1, NDOR1, TKFC, HACL1, FBXO33, FBXO31, IBTK, NFE2L2, ITGB1, COX4I1, CDC73, NADK2, SNAP29, BCAS2, ACTR3, ACTR2, VPS37C, ATRN, ARL4A, NDUFB9, SEC23A, C1S, NDUFB6, NDUFB2, MIEN1, TP53INP2, TP53INP1, CNNM4, NDUFA4, EIF3J, EIF3E, SLC25A36, EIF4A2, ERRFI1, TMEM41B, CCNH, ECI1, TXNDC16, IKZF5, CDC27, SEC62, TBC1D22A, PPA2, FBF1, ZMPSTE24, APOH, SARAF, APOB, PCK2, SURF4, SMARCA2, PAF1, INTS6, NDUFAF1, MRPS17, PTPRU, CSRNP2, HIBADH, MRPS12, PTPRK, SLC7A11, HYAL3, UIMC1, RAC1, AASS, CCT2, SEC13, NCOA4, CACYBP, NAPA, FAM172A, MON2, ORMDL1, VMP1, NEDD1, TFAP4, KLHL7, CCNYL1, ESYT1, FOXA2, PNPLA2, DPAGT1, MIDN, NARS, SLC30A5, DNAJB12, ADAM17, MASP2, TBC1D23, PSMD12, PSMD14, YTHDC2, LPXN, OST4, UBE2B, CCZ1, IGF2R, APPBP2, GALK2, CHD9, TNFAIP1, TMEM222, UFL1, MYO18A, MAN1A1, IFT57, NCBP1, GPCPD1, CHDH, NDUFS3, IRF9, RRN3, NAA25, ARFGEF2, LAMB2, U2SURP, CLCN7, GLB1, ERCC5, CYFIP1, VPS29, IPO11, SMC5, GCSH, RNF114, TFG, ADNP2, LUC7L, PIP4K2B, MPV17, RPL21, BIN3, PDXDC1, RPN2, CFI, LDHA, RRS1, NRBF2, ZNF142, JUND, BTG1, WDR48, SERPINA10, CLTB, TMEM189, MCEE, NDC1, AHSG, COQ10A, FAM210A, ZNF593, SGMS2, HSPA13, TM9SF3, RPS15A, EPCAM, SLC17A5, SF3B1, F10, SERPIND1, IL10RA, DEF6, LRPPRC, TM9SF2, NMNAT1, ABI3, ANKRD39, CLSTN3, LCLAT1, PPP1CB, ABHD13, IPO8, LAMP1, USP3, LEMD3, HIC2, PYGO2, ANKRD40, CSF1R, DNMT1, GLO1, CUL2, ABHD8, UBR2, PSAP, CD164, SEC16B, OSBPL7, TRAPPC5, COPS4, CNOT7, SYNJ1, COPS2, MAFF, TADA3, CNOT8, SERINC3, UFSP2, KIAA0100, OPA1, AP1G1, LBR, GPX1, AMT, PLRG1, PTP4A1, MCM5, DNAJB9, ASCC3, CUL4A, PSAT1, MFAP3, DNAJA2, RPS15, PDK1, SACM1L, MRPS18C, TLN1, STXBP3, TMTC4, SCFD1, PDCD10, CDT1, TMEM50A, HAL, RPS28, ANP32E, TMED7, AP3B1, UCHL5, SCNM1, NFX1, ATP6V0C, HAAO, AKAP8, NOL8, PDPR, METTL9, LYVE1, SPCS3, NR6A1, WASHC4, NDUFA10, TATDN1, TMEM14A, NSF, PDIA3, PHYH, ALG10, TRPC4AP, SELENOW, SELENOT, STAM2, DTX2, UBB, DNAJC19, CPSF2, DNAJC11, TBCD, TBCC, COQ5, COQ4, TBCK, BLMH, NFKBIB, USP36, DBT, FGA, HGSNAT, ABCC6, PPP2R5A, KDM3A, USP15, HLF, KDM3B, ETFDH, ATP1A1, HSPD1, GDAP2, KANSL3, XPNPEP3, SSBP1, PAXBP1

[1] MYOG, myogenin; CEBPB, CCAAT-enhancer-binding protein beta; USF1 and USF2, upstream stimulatory factors 1 and 2; JUND, junD proto-oncogene. [2] FET *p*-value, Fisher's exact test *p*-values. [3] FDR, false discovery rate.
